# Rapid methods including network meta-analysis to produce evidence in clinical decision support: a decision analysis

**DOI:** 10.1186/s13643-018-0829-z

**Published:** 2018-10-20

**Authors:** Øystein Eiring, Kjetil Gundro Brurberg, Kari Nytrøen, Magne Nylenna

**Affiliations:** 1 University of Oslo, Faculty of Medicine, N-0318 Oslo, Norway; 2 Norwegian Institute of Public Health, N-0403 Oslo, Norway; 3 The South-East Regional Health Authority in Norway, Postbox 404, 2303 Hamar, Norway; 4 Centre for Evidence Based Practice, Western Norway University of Applied Sciences, N-5020 Bergen, Norway

**Keywords:** Systematic review, Network meta-analysis, Rapid review, Clinical practice guidelines, Patient decision aids, Multi-criteria decision analysis, Bipolar disorder

## Abstract

**Background:**

Conducting systematic reviews is time-consuming but crucial to construct evidence-based patient decision aids, clinical practice guidelines and decision analyses. New methods might enable developers to produce a knowledge base more rapidly. However, trading off scientific rigour for speed when creating a knowledge base is controversial, and the consequences are insufficiently known. We developed and applied faster methods including systematic reviews and network meta-analyses, assessed their feasibility and compared them to a gold standard approach. We also assessed the feasibility of using decision analysis to perform this comparison.

**Methods:**

Long-term treatment in bipolar disorder was our testing field. We developed two new methods: an empirically based, rapid network meta-analysis (NMA) and an expert NMA, and conducted a patient survey. We applied these methods to collect effect estimates for evidence-based treatments on outcomes important to patients. The relative importance of outcomes was obtained from patients using a stated preference method. We used multi-criteria decision analysis to compare a gold standard NMA with the rapid NMA in terms of the ability of the gold standard NMA to change the ranking and expected values of treatments for individual patients.

**Results:**

Using rapid methods, it was feasible to identify evidence addressing outcomes important to patients. We found that replacing effect estimates from our rapid NMA with estimates from the gold standard NMA resulted in relatively small changes in the ranking and expected value of treatments. The rapid method sufficed to estimate the effects of nine out of ten options. To produce a ranking of treatments accurate for more than 95% of patients, it was necessary to supplement systematic with rapid methods and to use relative importance weights in the analysis. Integrating estimates of the outcome “treatment burden” had a larger impact on rankings than replacing rapid with gold standard methods. Using patients’ importance weights only modestly affected results.

**Conclusions:**

The transfer of knowledge to practice could benefit from faster systematic reviewing methods. The results in this preliminary assessment suggest that an improved rapid NMA approach might replace gold standard NMAs. Decision analysis could be used to compare evidence summarisation methods.

**Electronic supplementary material:**

The online version of this article (10.1186/s13643-018-0829-z) contains supplementary material, which is available to authorized users.

## Background

Bipolar disorder affects more than 2% of the world population [1, 2]. Long-term pharmacological treatment is generally recommended [3]. More than 40 treatments and 141 treatment outcomes are potentially relevant [4, 5].

Effect estimates from systematic reviews are crucial in different types of decision support for selecting treatment in bipolar disorder. In *clinical practice guidelines*, effect estimates are generally used to create *recommendations* on treatments, in bipolar disorder often ranked in two to four tiers [6–12].

*Patient decision aids* are tools designed to support patients and doctors in selecting the treatment likely to benefit the patient the most [13]. These tools are required to present the effect estimates of different treatment options *directly* [14].

*Multi-criteria decision analysis* (MCDA) provides support when the complexity of a decision surpasses the cognitive abilities of human decision-makers [15]. In MCDA, effect estimates are used to calculate the *expected values* and provide a ranking of the treatments [16]. The number of published MCDAs in health has increased steadily [17], fueled by a demand for more consistent, transparent and patient-centered approaches to decision-making [18, 19]. In the mental health field, at least 11 MCDAs have been conducted [20].

All tools depend on reliable effect estimates for the available treatments on important outcomes. Systematic reviews are the gold standard for identifying effect estimates, but the methodology is time-consuming and resource-intensive [21, 22].

Network meta-analyses (NMAs) based on systematic reviews can produce a larger body of evidence but consume even more time [23]. In bipolar disorder, frequent updating of the effect estimates for all main outcomes and treatments using gold standard methods is hardly feasible.

To address this problem, researchers have suggested several streamlining strategies for systematic reviews. Growing evidence suggests that many expedite methods can be conducted with relatively small implications on validity [24–27]. If rapid methods produce comparable results, then decision support tools might be more frequently updated and a larger number of conditions and patient-important outcomes addressed.

Substituting or supplementing gold standard methods is controversial and the consequences are insufficiently known [25, 27–31]. The impact of reducing scientific rigour when evaluating rapid reviews has typically been assessed along scientific measures such as validity.

In an era where patient-centeredness is the norm, we suggest an alternative way of estimating the consequences of reducing rigour when producing the evidence in decision support tools. *Using MCDA, the value of gold standard methods can be assessed based on their ability to change the expected values of treatments for individual patients, and the resulting ranking, compared to rapid methods*. To base this assessment on usefulness to individual patients, relative importance weights for the outcomes from each individual should be used in the analysis [32]. To further assess the value for clinical practice of the knowledge summarisation methods, the rankings produced by the MCDA can be compared to the rankings explicit in the tiers presented in clinical guidelines. This approach extends the scope of MCDA within healthcare. It also integrates patients’ evaluation of outcomes in the assessment of the methods.

We are not aware of any studies applying MCDA in the assessment of knowledge summarisation methods.

In the first step, we developed a rapid NMA and other expedite methods identifying effect estimates for treatments. We applied these methods to find effect estimates for all outcomes important to patients.

In the second step, we first elicited patients’ relative importance weights for all outcomes. We then compared the rapid NMA with a gold standard NMA, first in terms of methodology and then in terms of the impact on expected values and rankings of treatments, when results from the gold standard NMA replaced those from the rapid NMA. Next, we examined the impact when results from a patient survey and an expert NMA supplemented those from the gold standard NMA. In the last stage, we compared the results to rankings of treatment in clinical practice guidelines.

In the third step, we assessed the impact of using MCDA including patients’ relative importance weights in the comparison of knowledge summarisation methods and estimated what would constitute a requisite decision model.

## Methods

We used a value measurement type of MCDA to estimate the value of treatments in bipolar disorder and the value of systematic versus rapid methods [33]. MCDA is a subfield of operations research. To identify evidence that could be processed in this decision analysis, several substudies had to be conducted, applying qualitative and quantitative study designs and different statistical techniques.

### Step one: Identifying effect estimates for all outcomes important to patients on all relevant treatments

To identify effect estimates for all patient-important outcomes on all relevant treatments identified in this study within a relatively short time, we developed and applied several rapid strategies (Table 1). Together with the relative importance weights elicited in step 7, these data enabled us to construct a full decision matrix.

**Table 1:** Overview of methods used to identify effect estimates

Stage	Goal	Method
1	Identify patient-important outcomes	Systematic review, focus groups, self-explicated preference elicitation method.
2	Identify the treatment options	Options were identified in a rapid NMA and from a gold standard NMA.
3–6	Identify the effect estimates of all options on all outcomes	a. Empirically based rapid NMA for the outcomes “avoid acute manic episodes” and “avoid acute depressive episodes’. b. Tolerability rates for the outcome “side effects”, based on frequencies identified in a gold standard NMA. c. Patient survey for the outcome “avoid treatment burden” d. Expert NMA for the outcomes “avoid burden of manic symptoms between acute episodes” and “avoid burden of depressive symptoms between acute episodes”.

#### Outcomes important to patients

Which treatment outcomes are most important to patients with bipolar disorder when selecting long-term treatment? To answer this question, we first conducted a systematic review [34]. Because this review provided insufficient results, we conducted a separate study [5]. The objective of this study relevant to this paper was to construct a holistic taxonomy of patient-important outcomes intended for use in different decision-making contexts and decision tools. In this investigation, 22 outpatients from southern and eastern Norway participated in four focus groups. Only subjects with bipolar disorder type I or II, aged between 18 and 65 years and in a stable phase were included. Comorbid conditions and substance dependency were not reasons for exclusion. Participants were recruited by an open invitation on the Facebook site of the Norwegian patient association for people with bipolar disorder. They were also recruited by psychiatrists working in a psychiatric hospital in Oslo who invited patients to participate. The interview format in the focus groups was a structured group discussion. Participants were asked to imagine that they were going to assess and select long-term pharmacological treatment together with their clinician. They were then asked to consider what an ideal medicine should do and what would distinguish a good treatment from a bad one. Neutral, follow-up questions were asked, encouraging participants to detail their suggestions.

In a quantitative, second part of this study, relative outcome weights for 23 outcomes were elicited from all 22 participants. We applied the preference elicitation technique named the “self-explicated method” and presented the outcome ranges for all treatments [5, 35, 36].

#### Evidence-based treatment options

Options identified in a gold standard NMA [3] and in a rapid NMA (stage 3 in Table 1) were included in the analyses. Information on how the gold standard NMA was carried out was provided in the published paper and its appendix. In the gold standard NMA, the risk of bias was assessed using the Cochrane collaboration methods. Quality of evidence was assessed with the GRADE framework. Statistical heterogeneity was investigated using visual inspection of forest plots, supplemented using the *I*-squared statistic and tau. Consistency between direct and indirect sources of evidence was statistically assessed using computational and graphical tools with STATA version 13.0.23.

#### Avoid manic/mixed and depressive episodes

What are the effect estimates for manic/mixed and depressive episodes among patients with bipolar disorder? To answer this question, we first conducted a systematic overview of reviews of methodologies applied in rapid, systematic reviews. We then catalogued the methods described in the reviews, including their rationale, their empirical evidence and common shortcomings. Based on our overview, we defined strategies for maximizing validity, comprehensiveness, transparency and rapidity for a rapid NMA performed under strict time constraints.

Next, we applied these strategies in a rapid NMA to identify the effect estimates for medicines on preventing acute manic and depressive episodes in bipolar disorder. The quality of systematic reviews that were identified were assessed using AMSTAR [37]. Primary studies in the systematic reviews and those identified independently of reviews were excluded when they did not meet the eligibility outcomes. Inconsistencies were solved together with a third reviewer. We extracted data from the studies using a minimum data extraction template and performed an expedite NMA. Additional file 1 (pp. 1–11) provides more details about the rapid NMA.

#### Avoid side effects

What is the percentage of patients with bipolar disorder discontinuing treatment because of adverse effects, for each treatment? To answer this question, we did not perform research ourselves. The gold standard NMA contained risks for discontinuation due to adverse effects, for all treatments in the NMA. We converted these risks into absolute risks for each treatment, using absolute risks for patients receiving placebo as the baseline.

#### Avoid treatment burden

What is the treatment burden for each treatment, expressed as a degree of burden on a 0 to 100 scale? Effect estimates for treatment burden, an outcome found in our earlier work to be important to patients, was lacking in the rapid NMA. To answer the question, we developed descriptions of the treatment burden for all relevant treatments using information from Summaries of Product Characteristics [38] and Micromedex © [39]. We modeled all descriptions applying the framework described in Tran et al. [40] depicting the major constraints, arrangements and recommended actions relevant for each medicine. Nineteen of the 22 patients having participated in the taxonomy study had responded that they were interested in participating in further work and were invited by one author (KN) to participate in this substudy. The participants, all with either bipolar disorder type I or II, replied to a questionnaire with descriptions for all relevant treatments [5]. Participants first evaluated the burden common to all medicines and then the additional burden regarding each specific medicine.

#### Avoid manic and depressive burden between episodes

What is the manic and depressive burden between episodes, for each treatment? Effect estimates for symptoms and consequences of depression and mania between episodes were also lacking in the gold standard NMA. To answer this question, we piloted the use of NMA in expert opinion. A questionnaire was piloted with three psychiatrists, revised, and sent to a convenience sample of 42 physicians with experience in treating patients with bipolar disorder. Invitations were sent by email and respondents completed an online questionnaire. Participants were asked to estimate the percentage of their patients who experienced mild or moderate depressive or manic symptoms most of the time between acute episodes, for all treatments included in the NMA. In the analysis, the dataset from each clinician was regarded as an individual trial. To be able to compare with the gold standard approach, physicians also assessed the percentage experiencing acute episodes.

### Step two: Impact on expected values and rankings of treatments of rapid and gold standard methods

#### Individual, relative importance weights

What are the individual relative importance weights for each treatment outcome important to patients? A dataset with weights was necessary to complete the decision matrix. To answer this question, we developed comprehensive descriptions of the six outcomes based on patients’ accounts of common features and consequences of treatments in focus group interviews. A questionnaire was sent to a convenience sample of forty-four patients recruited from the website of the Norwegian patient organization for people with bipolar disorder. Nineteen patients had participated in the taxonomy and treatment burden study, and 26 patients were recruited after a new invitation on the website. A self-explicated stated preference exercise elicited the participants’ relative importance weights for the outcomes. The expected performances of the best and the worst option for each outcome were presented during this trade-off.

#### Basic comparison of rapid and systematic NMA

How do the methods and results in the rapid NMA compare to the methods and results in the systematic NMA? Although the overall comparison method of rapid and systematic NMAs in this paper is MCDA, we provide a methodological comparison for illustrative purposes. Contrasting with a gold standard approach, our rapid NMA was designed to identify and include pre-appraised evidence from primary studies in quality-assessed systematic reviews, supplemented with primary studies identified and assessed in the McMaster Knowledge Refinery; this strategy replaced the gold standard search for primary studies. Furthermore, only easily retrievable abstracts and full-text, electronic sources were considered. AMSTAR criteria were applied to systematic reviews, and principles from the Cochrane collaboration were used to assess the risk of bias in individual studies. Neither inconsistency nor intransitivity was explored in the rapid NMA. These less rigourous approaches were selected in accordance with the overall goal of this work.

In addition to the methodological comparison, we provide a comparison of the findings in the rapid NMA and the corresponding findings in a traditional gold standard NMA, published 1 month after completion of the rapid NMA [3]. AMSTAR and PRISMA scores [41] and risk of bias assessments were also compared.

#### Expected values and ranking of treatment options

What are the expected values and corresponding ranking of treatments, when datasets from systematic reviews replace those from rapid reviews or are supplemented with datasets from patient-important outcomes not included in the systematic review? How do the expected values and rankings obtained from different datasets compare to each other? To answer these questions, we performed a number of MCDAs, either replacing or supplementing the results from the gold standard review. Expected values, area under the curve (AUC—a measure of overall rank) and the corresponding rankings of the options were calculated by applying three different approaches. In the first approach, we performed an MCDA with the dataset from an older NMA [42], providing episode rates, and then replaced this dataset with datasets from the rapid and then the gold standard NMAs. Comparing the results of these three MCDAs, we identified changes in expected values, rankings and AUCs (Table 2). We limited this analysis to treatments included in all three NMAs. Second, we performed an MCDA with a minimum number of outcomes and then supplemented the estimates from the gold standard review with estimates from a larger number of outcomes, identified by us, in a stepwise manner. We investigated the impact of including more outcomes on expected values, rankings and AUCs (Table 3).

**Table 2:** The effects of replacing the dataset from an old NMA (Vergel) with datasets from a rapid and then a gold standard NMA (Miura)

Analysis	Episode rates	Side effects	Treatment burden	Symptoms between episodes
1.1	Vergel			
1.2	Rapid			
1.3	Miura			
1.4	Vergel	X		
1.5	Rapid	X		
1.6	Miura	X		
1.7	Vergel	X	X	
1.8	Rapid	X	X	
1.9	Miura	X	X	
1.10	Vergel	X	X	X
1.11	Rapid	X	X	X
1.12	Miura	X	X	X

**Table 3:** The effects of including an increasing number of outcomes

Included outcomes				
Analysis	Episode rates	Side effects	Treatment burden	Symptoms between episodes
2.1	X			
2.2	X	X		
2.3	X	X	X	
2.4	X	X	X	X

In the MCDAs, we applied the simple weighted sum equation in the statistical software R (The R Foundation for Statistical Computing, Austria, version 3.4.1) [43]. We calculated the expected values of the options using:

$$ \mathrm{Expected}\ \mathrm{value}={\sum}_{i=1}^n{w}_i{p}_i $$

where *w*_*i*_ is the relative importance weight for the *i*^th^ outcomes and *p*_*i*_ is the probability or expected magnitude of an outcome. All *p*_*i*_ were calculated as the product of absolute baseline risk and risk ratios. In each MCDA, we calculated the ranking of all available treatments resulting from the expected values and the surface under the cumulative ranking curve (SUCRA or “area under the curve”—AUC).

The impact of the four different MCDA models was evaluated based on mean and individual expected values of the medicines, differences between those values, and changes in means when replacing one dataset with another. In accordance with the patient-oriented focus of this work, we aimed for maximum granularity in the analyses. We identified the top, middle and lowest ranked treatments based on the AUC, the changes in ranks resulting from using a new dataset, and variation in the rank of the treatments for individual patients, dependent on the dataset. The ability of an approach to produce stable rankings for a maximum amount of patients, distance of a treatment’s expected value to the value of placebo and changes in the AUC were also used to evaluate the methods.

All MCDA analyses were performed in accordance with the ISPOR MCDA Good Practice Guidelines Checklist [32].

#### Comparison to recommendations in clinical practice guidelines and textbooks

How do the rankings obtained in the analyses compare to rankings in clinical practice guidelines and online textbooks? To answer this question, we conducted a search for current clinical practice guidelines and textbooks containing recommendations for long-term treatment in bipolar disorder and identified the outcomes used and the rankings of the options, placed in tiers, in each resource. We then compared the outcomes and rankings in the guidelines with those in the MCDAs.

### Step three: The impact of using patients’ relative importance weights in the comparison of knowledge summarisation methods

#### Impact of individual importance weights

What is the impact on expected values and rankings of treatments in the MCDAs when average relative importance weights are replaced with those from individual patients? To answer this question, the same methods were used as described in step 2, stage 9: “Expected values and ranking of treatment options” section (Table 4).

**Table 4:** The effects of replacing average importance weights with individual patients’ weights

Analysis	Importance weights and ratings				
	Impor-tance weights	Episodes	Side effects	Treatment burden	Symptoms between episodes
3.1	Average	X			
3.2	Individual	X			
3.3	Average	X	X		
3.4	Individual	X	X		
3.5	Average	X	X	X	
3.6	Individual	X	X	X	
3.7	Average	X	X	X	X
3.8	Individual	X	X	X	X

We performed an MCDA for three individual patients with very different relative importance values and compared the expected values and rankings of treatments for the three patients (details provided in Table 5).

**Table 5:** Expected values and rankings of treatments for three patients

Analysis	Episode rates*	Side effects	Treatment burden	Symptoms between episodes*
1 M, 2D, 3S*	Miura	X	X	X

The effects of replacing the rapid with a gold standard dataset, for three patients prioritizing manic (1 M), depressive (2D) and side effect/treatment burden (3S), respectively. *One analysis per each of the three patients

#### A requisite model

What is a requisite approach for estimating expected values and the corresponding rankings of treatment options in bipolar disorder? A requisite model is an overall measure comparing the rapid and the gold standard methods and an estimate of which combination of approaches is requisite. To answer the question, we defined a requisite model and compared the analyses to this model. A requisite model was defined as one including all options found among the top two thirds of treatments for at least 95% of patients and all outcomes able to change the treatments included among the top three treatments for more than 5% of patients. A knowledge summary method was defined as requisite in this model if it resulted in the same top, middle and bottom ranking of the options when an increasing number of outcomes were included. We also required that the mean expected values of the treatments, resulting from rapid methods, should not differ more than 1% compared to the gold standard method. We also defined that using individual relative importance weights to compare gold standard and rapid methods would be required in the model if the use of average importance weights resulted in major changes in the rankings for more than 5% of the patients.

## Results

### Step one: Identifying effect estimates for all outcomes important to patients on all relevant treatments

#### Outcomes important to patients

Twenty-two patients participated in the focus groups and completed an exercise eliciting their relative importance weights [5]. Sixty-four percent were women, the mean (SD) age was 42 [12] years and the mean (SD) years since diagnosis was eight [6]. Fifty-nine percent had bipolar disorder type I and 41% type II. Fifty-nine percent mostly experienced depressive episodes and 27% manic episodes. Ninety-one percent were in a neutral mood at the time of participation. Lithium (36%), lamotrigine (36%) and aripiprazole (23%) were the most frequently used medicines. Six composite outcomes were constructed: (1) avoid manic episodes, (2) avoid depressive episodes, (3) avoid manic burden between episodes, (4) avoid depressive burden between episodes, (5) avoid side effects and (6) avoid treatment burden. Details are provided in a previously published article [5].

#### Evidence-based treatment options

Seventeen treatments and treatment combinations, including no treatment, were identified in the gold standard NMA [3] and 16 in the rapid NMA (Additional file 1, pp. 12–13).

#### Avoid manic/mixed and depressive episodes

Six reviews were included in our overview of rapid reviewing methodologies [25, 27–31]. We selected 21 strategies to be used in a rapid NMA. The majority of strategies addressed common deficits in rapid reviewing, or there was evidence for no or acceptable loss of validity when replacing gold standard methods (Table 6). A core strategy was supplementing a search for systematic reviews with searching for primary studies in the database McMasterPlus [44].

**Table 6:** Rapid review strategies. The summary of the rationale and empirical evidence for selecting each of the strategies is available on request

Rapid review strategies	
1. Perform the rapid NMA in a well-established field and for an efficacy or effectiveness question.	
2. Use an a priori, non-iterative approach. Define a clear and narrow question using the PICO format. Do not adjust the conceptual framework nor the search terms during the review process.	
3. If appropriate, limit the context, for instance to primary healthcare or a specific geographic area.	
4. Narrow the search criteria through consultation with experts.	
5. Assemble a team with experience in conducting systematic reviews. The team, as a minimum, should consist of a clinician, a methodologist with expertise in systematic reviews, and a librarian. Team members must have enough time allotted when beginning the review process.	
6. Search for systematic reviews and overviews of reviews first.	
7. Apply date restriction through consultation with experts and use a strict cutoff date for article retrieval. For overviews of reviews, restrict the search period to the last few years.	
8. Apply English as a language restriction.	
9. As a minimum, search in MEDLINE, Embase, Central and CDSR.	
10. Do not search for grey literature.	
11. Include only readily accessible published literature. Exclude studies lacking an electronically available abstract or full-text article.	
12. Do not search in non-electronic sources.	
13. Consult with experts about missed articles.	
14. Hand-search reference lists in the included papers.	
15. Report all methods used including the search strategy, according to PRISMA.	
16. Include two reviewers at all stages, or alternatively use a second screener to check unclear or excluded citations.	
17. Report strategies used to deal with discordance between reviewers.	
18. For systematic reviews and overviews of reviews, assess the methodological quality of the reviews using AMSTAR to determine whether to include the studies in the review or not.	
19. If high-quality systematic reviews exist, then limit the search for primary studies to those published after the most recent systematic review.	
20. Only include RCTs that have been appraised in an included systematic review, or that have been quality-assessed in the McMaster Knowledge Refinery or a similar process.	
21. Map the studies and interventions. Use a minimum data extraction sheet, extract data from the individual studies, and perform an expedite network meta-analysis.	

**Fig. 1:**
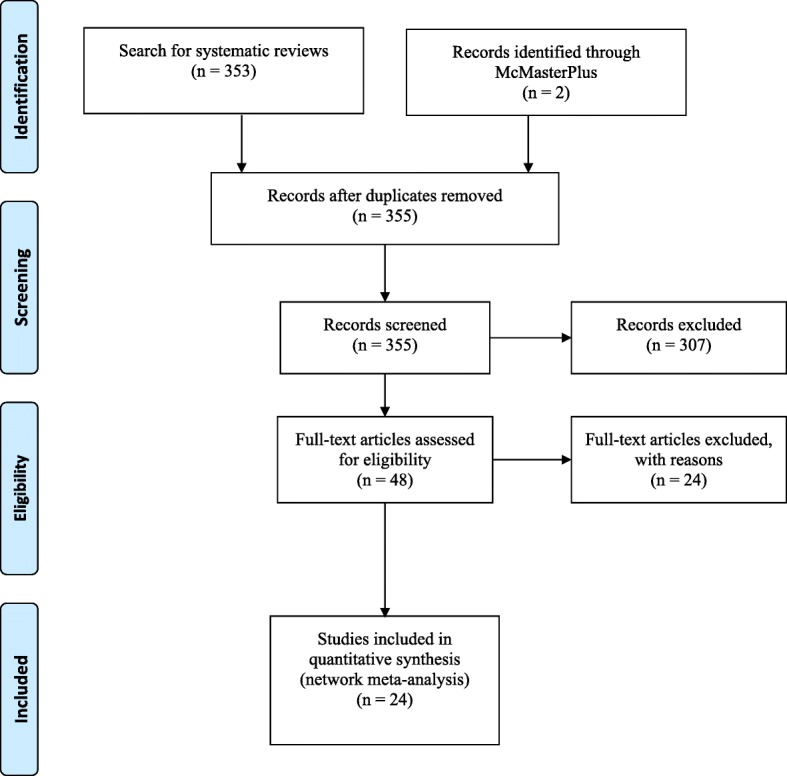
PRISMA flow diagram for the rapid network meta-analysis

Guided by the selected strategies, we conducted a rapid NMA. Three hundred fifty-five papers were identified and 48 citations retrieved in full text. Results from 24 primary studies with 3632 patients were included. Twenty-two studies were retrieved from the systematic reviews and two from the McMasterPLUS database. Figure 1 presents the PRISMA flow diagram.

The relative episode risks for treatments identified in the rapid NMA are presented in Additional file 1, pp. 12–13).

#### Avoid side effects

In the gold standard NMA, 69–98% of patients, dependent on medicine, did not discontinue their treatment due to adverse events during a mean study duration of 74 weeks [3].

#### Avoid treatment burden

Eighteen patients completed our survey. Sixty-seven percent were women, and the mean (SD) age was 41 (9) years. On a 0–100 scale, where 100 represented maximum treatment burden, risperidone LAI was reported to be the most burdensome treatment (70.7), whereas valproate was least burdensome (42.7) (Additional file 1, p. 14).

#### Avoid manic and depressive burden between episodes

Ten of the 42 invited physicians (24%) answered the questionnaire (Additional file 1, pp. 15–18). Ninety percent were men, and the mean (SD) experience in treating people with bipolar disorder was 25 (8) years. The experts estimated that valproate + aripiprazole, risperidone and lithium+valproate were most effective against manic symptoms between episodes and imipramine, valproate+lamotrigine and lamotrigine most effective against depressive symptoms When their rank orders of treatments regarding acute episodes were compared to those from the gold standard NMA, there was no significant correlation (Additional file 1, pp. 19–20)

### Step two: Impact on expected values and rankings of treatments of rapid and gold standard methods

#### Individual, relative importance weights

Twenty-eight patients (response rate 64%) provided their relative importance weights for all outcomes. Sixty-eight percent were women, the mean (SD) age was 44 (9) years, 46% had bipolar disorder type I and 46% had type II. Sixty-four percent predominantly experienced depressive and 22% manic episodes. On average, patients reported that avoiding severe depressive and severe manic episodes were the most important outcomes. Avoiding mild and moderate manic episodes and avoiding treatment burden was least important (Additional file 1, p. 20).

#### Basic comparison of rapid and systematic NMA

A less rigourous approach in the rapid versus the gold standard NMA was found for 11 out of 27 PRISMA and four out of 11 AMSTAR requirements. Twenty-four studies were included in the rapid NMA, compared to 33 in the gold standard NMA. The rapid NMA identified eight of the nine non-placebo treatments included in the main closed-loop network in the gold standard NMA. Main reasons for discrepancies between the rapid and the gold standard NMA were recent studies missing in the McMaster Plus service, stricter quality outcomes in the rapid NMA and different inclusion criteria (Additional file 1 including the PRISMA checklist, pp. 21–27).

#### Expected values and ranking of treatment options

The datasets for all results summarised below are available on request. Effect estimates from our work either replaced or supplemented those from the gold standard NMA.

a. Impact of replacing rapid with gold standard estimates. In the first set of analyses, the datasets from an older, a gold standard and the rapid NMA were used in MCDAs, applying an increasing number of outcomes (Table 2). Only the gold standard NMA included an exploration of intransitivity and inconsistency.

First, we replaced effect estimates for manic and depressive episodes in the rapid NMA, with estimates from the gold standard NMA, in the analysis. The same medicines were found in the top three, the middle two and the bottom two ranked options for these two variants. Contrasting with this finding, the medicines in the three tiers resulting from the dataset in the older NMA differed from those in the gold standard and the rapid NMA. The rank orders were based on AUC. The absolute AUC intervals inter-tiers between the rapid and the gold standard NMA were 0.244 and 0.274, respectively (episodes only).

Based on the mean AUCs for episode rates only, we identified the top three treatments overall, for the gold standard and the rapid NMA. Seven percent and 5% of the treatments appearing among the top three options for the individual patients were not among those resulting from the gold standard and the rapid NMA.

**Fig. 2:**
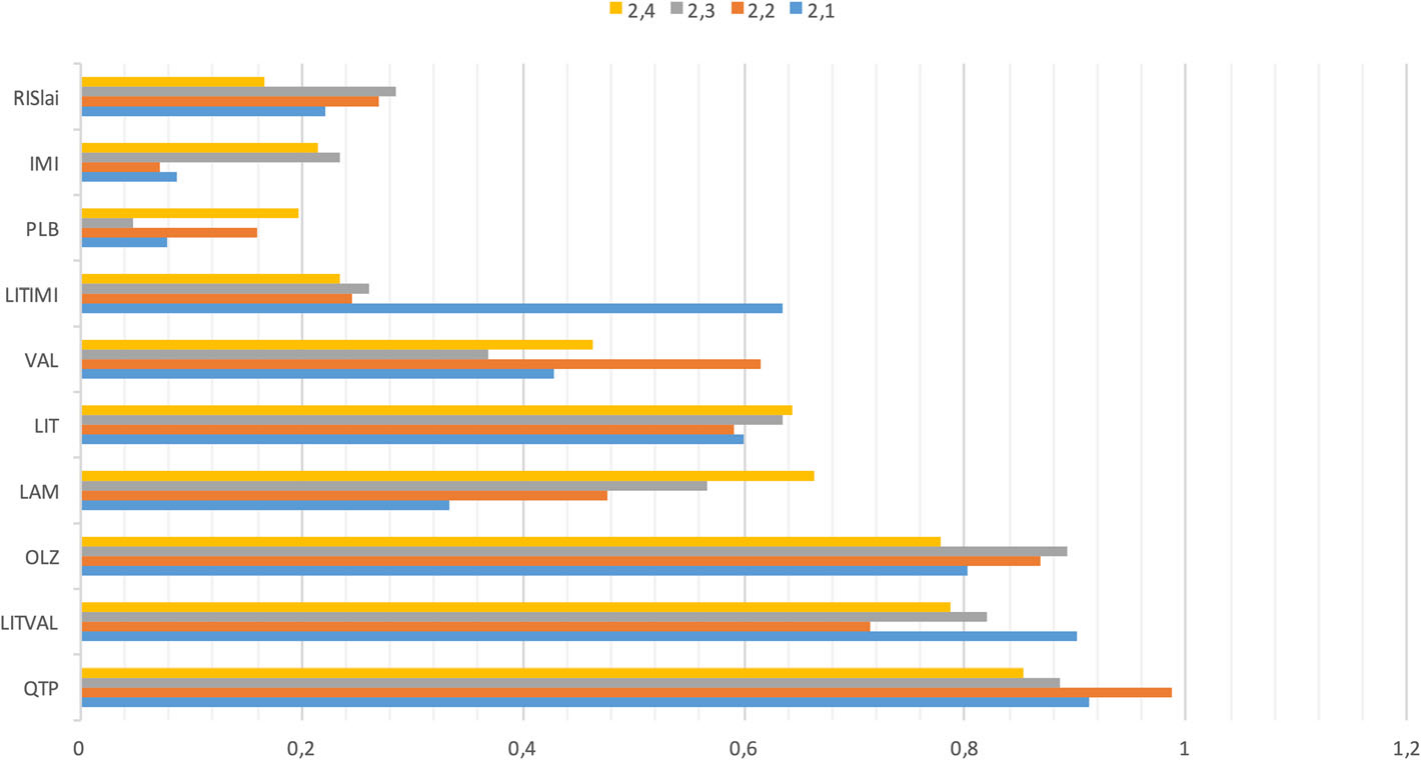
AUCs for the four different analyses, per treatment. Note that the ranking of lamotrigine increased for each added outcome

Using episode rates only, the mean expected values for all medicines overall—old, rapid and gold standard results—were 83.4%, 81.4% and 80.2%. When including four outcomes, the mean expected values in the rapid and gold standard NMA were reduced to 80.0% and 79.1%. The mean expected value in the gold standard NMA now differed 3.2% from the old and 1.2% from the rapid NMA. The mean difference in AUCs for the treatment options between the rapid and the gold standard NMA was 0.08.

b. Impact of increasing the number of outcomes. In these analyses, estimates from the rapid methods supplemented those from the gold standard approach for the three outcomes not addressed in the gold standard NMA (details provided in Table 3). One of the treatments included in the top three, middle four and bottom three-tier changed when estimates for side effects were added, and one when treatment burden was added. The order of the first three options was (1) quetiapine, (2) lithium+valproate and (3) olanzapine. The rankings were based on AUCs (Fig. 2).

The number of medicines ranked first, second or third by at least 5% of patients increased from 4 to 5, 6 and 8 as the number of outcomes was increased; only risperidone LAI was never among the top three (Table 7).

**Table 7:** Rank for 28 patients at the ten positions available, when all outcomes were included in the analysis

	Rank 1	Rank 2	Rank 3	Rank 4	Rank 5	Rank 6	Rank 7	Rank 8	Rank 9	Rank 10
PLB	0	2	0	0	0	1	6	5	2	12
OLZ	6	10	5	1	1	1	4	0	0	0
LIT	0	0	2	20	4	2	0	0	0	0
RISlai	0	0	0	0	0	2	6	4	8	8
LAM	5	3	0	4	11	4	1	0	0	0
LITVAL	6	5	10	1	4	2	0	0	0	0
QTP	11	7	7	0	1	1	0	1	0	0
VAL	0	0	3	2	4	11	4	4	0	0
IMI	0	1	1	0	0	1	3	8	10	4
LITIMI	0	0	0	0	3	3	4	6	8	4

*PLB* placebo, *OLZ* olanzapine, *LIT* lithium, *RISlai* risperidone LAI, *LAM* lamotrigine, *LITVAL* lithium + valproate, *QTP* quetiapine, *VAL* valproate, *IMI* imipramine, *LITIMI* lithium + imipramine

Lithium monotherapy was ranked number five in all four AUC analyses. 7.1% of patients ranked lithium among the three best options.

When the two disadvantages of medicines were included in the model, 7% of patients ranked placebo among the three best options. The difference between the mean expected value of all medicines together and the expected value of placebo was reduced from 7.0%, when only episode rates were considered, to 1.7% when side effects and treatment burden was included. Adding estimates for the outcome “treatment burden” to the analysis reduced the mean expected value per medicine with 3.6%.

#### Comparison to clinical practice guidelines and textbooks

We identified six clinical practice guidelines and textbooks [6–12]. Forty treatments and treatment combinations were presented in the resources, ranging from 8 to 25 per guideline, compared to the 10 research-based options included in this review. All sources recommended lithium as a first-line option. None of the resources included a comprehensive treatment burden outcome. Details available on request.

### Step three: The impact of using patients’ relative importance weights in the comparison of knowledge summarisation methods

#### Impact of individual importance weights

When individual importance weights replaced average importance weights, the top three options remained identical, both when only episode rates and when all outcomes were included. Using individual importance weights and only episode rates, 6% of all top three ranks from individual patients were outside of the top three ranks resulting from means. When we included all outcomes, 20% of all individual top three ranks regarded four treatments not among the top three resulting from averages. When the second, third and fourth dataset was applied, a total of nine treatments had to be included among the top three to accord with all patients’ importance weights. The absolute difference between the means of the first three, middle two and bottom two options based on AUC was 6.6 and 8.1% (Table 4).

Three individual patients were selected among the 28 to illustrate the impact of specific preference patterns. The first patient assigned high importance to avoid manic episodes and symptoms. For this patient, the top three options were olanzapine, lithium+valproate and lithium. The second patient preferred avoiding depressive episodes and symptoms, and with these preferences, lithium+valproate was suggested as the first option, followed by olanzapine and then lamotrigine. For the patient who prioritized to avoid side effects and treatment burden, lamotrigine, placebo and lithium resulted as the top three options. The absolute difference in expected value between the option ranked first and third was 1.0 to 1.9% for the three patients. All six outcomes were used in the analysis.

#### A requisite model

To identify the top two thirds of treatments for at least 95% of patients, all options had to be included. All six outcomes were necessary to identify changes in the top three options for at least 95% of patients. The expected values resulting from the rapid NMA approach differed less than 1% from the gold standard, and the ranking was stable between the two approaches. However, the rapid NMA approach did not identify all relevant options and this approach was thus not required without further improvement. Using averages led to misleading results for 20% of patients and applying individuals’ relative importance weights was necessary for the approach to be requisite.

## Discussion

### Key findings

The main methods developed were an empirically based rapid NMA and an expert NMA. Using a combination of the two NMAs, pre-assessed evidence and a patient survey, we identified effect estimates for long-term treatments in bipolar disorder on six patient-important outcomes. Second, using these datasets, patients’ relative importance weights for the outcomes were elicited, taking the best and worst alternative into account. Third, a multi-criteria decision analysis was performed on datasets where elements in this knowledge base replaced or supplemented datasets from a gold standard NMA to estimate the impact of these variations. It was meaningful to assess the relative value of performing gold standard versus rapid evidence summarisation.

Replacing estimates from a rapid NMA with those from a gold standard NMA did not cause significant changes in the ranking of medicines when patients’ relative importance weights were applied. The average, expected value of treatments differed less than 1 % between the rapid and the gold standard datasets. However, the rapid NMA missed an option included in the gold standard NMA.

The incorporation of treatment burden into the analysis had a much greater impact on the expected value of the treatments than replacing rapid NMA with gold standard NMA effect estimates. This reflected the results; patients’ relative importance weights and their estimates of treatment burden varied substantially. However, the impact of incorporating relative importance weights on the ranking of treatments was modest.

For the decision model to be requisite, it was necessary to supplement the estimates from the gold standard NMA with estimates from the rapid methods. All ten research-based treatments, estimates for the six patient-important outcomes and the patient’s relative importance weights had to be included. Each added outcome increased the variation in the ranking of treatments among patients.

### Meaning of study

With small improvements, producers of implementation tools such as clinical practice guidelines and patient decision aids could benefit from using rapid methods to identify evidence to be used in the tools. Rapid methods have advantages compared to gold standard methods both in terms of time and resource use and in terms of how many patient-important outcomes can be addressed in the tool.

As an example, although the importance weights of “treatment burden” were generally low, including effect estimates for this outcome had a greater impact on the ranking of treatments for individual patients than replacing rapid with gold standard NMA effect estimates. Including effect estimates on treatment burden reduced the expected value of medicines. The differences in expected values between medicines and placebo were also reduced. Including this outcome therefore favoured “medicine-free treatment” compared to medication option.

Both the outcome “treatment burden” and the treatment option “medicine-free treatment” are often omitted in clinical guidelines and online textbooks on long-term treatment in bipolar disorder [6–12]. This negligence parallels the finding that psychiatrists assign less importance to side effects than patients [34]. Our study suggests that the burden that can be expected from treatments should be included in implementation tools so that this outcome can be part of patient-clinician discussions.

This study underlines the need for implementation tools. Although the mathematical analyses performed to provide the rankings in this study were simple, they surpass the cognitive ability of humans. This is also due to the sheer amount of data, which can only be expected to increase. To achieve the ideal of evidence-based medicine, which is to base the selection of treatment on an integration of all available evidence with the individual’s preferences, clinicians need to supplement their clinical judgment with implementation tools. The effect estimates in this MCDA are already used in decision support systems providing a personalized ranking of treatments [45, 46].

### Results in context

Using patients’ outcomes and relative importance weights in MCDAs addressing interventions to be implemented in healthcare is not common. Although at least 66 MCDAs have been performed in this field, only 12 applications have been reported to address healthcare interventions per se [19]. Second, out of ten MCDAs included in a different overview of MCDAs in healthcare, six did not involve patients when identifying relevant treatment outcomes, whereas patients were included to some degree in the remaining four [47](Additional file 1, p. 28). In at least seven out of the ten analyses, only group averages were used as importance weights.

At least one MCDA on pharmacotherapy in bipolar disorder has been performed before. This study obtained relative importance weights and performance ratings uniquely from pharmacists [48]. A composite burden of treatment outcome was not included. The analysis concluded that lithium had the highest total value score in long-term treatment. This expert-based conclusion, using on a limited number of outcomes, contrasts with the mediocre ranking of lithium produced in our analyses.

This study is also more comprehensive than most MCDAs in healthcare. NMAs, applied to two datasets in this material, maximizes the number of outcomes that can be addressed. We identified only one other published MCDA in healthcare, producing and using data from an NMA [49].

This work appears to be the first MCDA to evaluate the gain of using gold standard NMA methods compared to rapid NMA methods. Our results suggest that the incremental value of more rigourous knowledge summary methods might be meaningfully estimated not only based on traditional scientific standards but also from the extent the methods can be expected to change the expected values and ranking of treatments for individual patients.

### Limitations

There is considerable uncertainty surrounding the estimates used in our analyses. For instance, in the gold standard NMA, the quality of the evidence was low or very low for all except two options. However, this NMA is still the most updated and systematic one available for bipolar maintenance treatment.

To address all important outcomes, pragmatic adjustments were applied. For instance, there were four symptoms and episode outcomes, and two outcomes addressing the disadvantages of medicines—side effects and burden of treatment. This imbalance possibly favoured medicine over placebo [50]. The numeric range of the estimates varied from one outcome to another; however, patients had access to those ranges during the trade-off exercise and were asked to take them into regard.

All outcomes were composed of several sub-outcomes. If the relative importance of the sub-outcomes had been elicited directly, then more specific valuations would have been produced. However, the burden on the respondents would have been multiplied, as well as the time needed to search for effect estimates.

The definition of the side effects outcome, equaling it with tolerability rates, can be expected to produce estimates that favor medicines over placebo, compared to for instance using the percentage of patients experiencing side effects.

In the rapid NMA subproject, the appropriateness of the transitivity and consistency assumptions were not estimated, and GRADE assessments were not performed. Inconsistencies per loop were not accessed and may exist. Consequently, the numerical results from the rapid NMA presented in this manuscript should be interpreted with caution. However, performing a gold standard systematic review and NMA was not an objective of the study. Instead, methodological rigour was consciously reduced, and the consequences of this reduction assessed in terms of how the results impacted treatment rankings for individual patients.

Sparse evidence currently exists on how to conduct MCDAs in healthcare, and the design and execution of these methods are likely to change in the future [17, 51].

### Implications for research

First, this study provides early and tentative evidence that rapid NMAs might produce results comparable to those in gold standard NMAs. However, the inability of our search strategy to identify primary studies published after the search date in the most recent systematic review resulted in one missing treatment option. To improve this shortcoming, we suggest that the rapid NMA approach is improved with for instance the use of Clinical Queries in PubMed, the “related articles” function in PubMed or the “cited by” in Google Scholar and that this approach is evaluated in research. Future improvements also could include a global inconsistency test, with rapidity being maintained performing one global test as an alternative to performing many loop inconsistency tests [52].

Second, the common finding that expert opinion might not correspond with research findings was also found in this work. Possibly, better elicitation techniques could increase the quality of clinicians’ estimates in an expert NMA. Alternatively, it might be the case that in some instances, the average clinician cannot reliably estimate the quantitative effect of treatments even though the clinician is familiar with the treatments. This open and clinically relevant question could be examined in further research.

Third, the outcome “burden of treatment” was found to particularly affect the ranking of treatments. Better methods for measuring this burden should be developed [53].

### Implications of the work

This study highlights the need for transparency regarding evidence and preferences implicitly or explicitly included in implementation tools. As an example, the mediocre ranking of lithium consistently found in these analyses contrasts with the high rank given to lithium in most clinical guidelines and online textbooks. Possibly, the implicit relative preferences used to produce the rankings differ from those of individual patients. For rankings to be in accordance with the personal priorities of patients, rankings of treatments should be based on the importance weights from the individual patient. A possibility to integrate these weights is currently not a feature in clinical guidelines. Supplementing clinical guidelines with MCDAs would address this deficit.

The relatively small numbers of participants included in the rapid methods reduce the generalizability of the results. However, producing rankings and expected values that are as accurate as possible for the individual patient is not only a question of larger patient groups but also a question of identifying the individual patient’s importance weights and estimates that can be elicited from the individual, such as “treatment burden” and former experience with treatments.

Thus, any analysis based on effect estimates from groups can be regarded as crude approximations in terms of relevance for the individual patients and improvement of the knowledge elicitation methods will not change this condition. Rather, this limitation should be addressed with better technologies.

An MCDA-based system that has been developed integrates the estimates in this study with importance weights and estimates from the patient, including the individual patient’s self-reported treatment results. The uncertainty regarding each estimate is also integrated, producing a continuously updated ranking of the treatments and their expected values, for each individual [54]. From the individual’s point of view, personalization of effect estimates and rankings is arguably more relevant than generalization. This should be taken into account when producing implementation tools.

## Conclusion

It was feasible to conduct a rapid systematic review and network meta-analysis to produce effect estimates replacing estimates from a gold standard systematic review and network meta-analysis. Using decision analysis to compare rapid and gold standard methods was feasible and meaningful. With modest improvements, rapid network meta-analysis might replace gold standard network meta-analysis. Authors of implementation tools might use an improved network meta-analysis method to reduce the time and resources needed to transfer knowledge to practice and to create more patient-oriented tools.

## Additional file

Additional file 1: This file contains nine appendices providing further detail on the methods and results of the study. (DOCX 239 kb)

## Abbreviations

AUC: Area under the curve
MCDA: Multi-criteria decision analysis
NMA: Network meta-analysis
SUCRA: Surface under the cumulative ranking curve
