# Supplementary material for: Rapid methods including network meta-analysis to produce evidence in clinical decision support: a decision analysis
Source: Syst Rev. 2018 Oct 20;7:168. doi: 10.1186/s13643-018-0829-z (PMC6195718; doi:10.1186/s13643-018-0829-z)
Supplement: Supplementary file 1 — This file contains nine appendices providing further detail on the methods and results of the study. (DOCX 239 kb) [file 13643_2018_829_MOESM1_ESM.docx]

**Appendices**

Appendix 1 – 4 all concern *stage one*: identifying effect estimates for all outcomes important to patients. Subparts a – f in Appendix 1 provide details on how the rapid review was conducted.

Appendix 5 – 7 concern *stage two*: the impact on expected values and rankings of treatments of rapid versus gold standard methods. Appendix 8 provides a descriptive comparison of the methods.

Appendix 9 is a simple overview of features in current multi-criteria decision analyses summarised in the discussion part.

**_______________________________________________________________________________________**

**Stage one: Identifying effect estimates for all outcomes important to patients_____________________________________________________________________________**

**Appendix 1: Rapid NMA details**

a. Search strategy for systematic overview of reviews of methodologies applied in rapid, systematic reviews

*Bibliographic databases*

- Cochrane Library (Cochrane Reviews, Other Reviews, Trials, Methods Studies og Technology Assessments)
- PubMed/Medline
- Embase
- Cinahl
- PsychInfo
- LISTA
- Google Scholar

*Grey literature databases*

- Websites of international health organisations and methodology centres
- Core journals (International Journal of Technology Assessment in Health Care, Health Research Policy and Systems)
- Conferences & experts

*Keywords/text-terms*

‘RR$’

ultra ADJ1 ‘RR$’

‘Scope article$’

‘Systematic review$’ ADJ2 (rapid OR short OR mini)

‘evidence summar$’ NY

‘evidence synthes$’ NY

synthesis ADJ2 knowledge NY

‘assessment report$’

‘rapid assessment report$’

technote$

technology ADJ2 overview$ NY

‘health technolog$’ ADJ2 assessment$ NY

HTA$ NY

‘health impact assessment$’ NY

*Thesaurus terms/Controlled vocabulary*

- Evidence-Based Practice (Embase, PsychINFO)
- Methodology (Embase, PsychINFO)
- Review Literature as Topic (Cochrane, Medline)
- Research Methodology (Cinahl)
- Literature Review (Cinahl)

Text word/keywords where searched in the Title, Abstract and keywords part of the article indexed. We piloted the use of keywords in the search strategy and performed several iterations. In the search strategy a majority of non-thesaurus terms were applied (text-words).

b. Flowchart for how studies were identified in the systematic overview of reviews of methodologies applied in rapid, systematic reviews


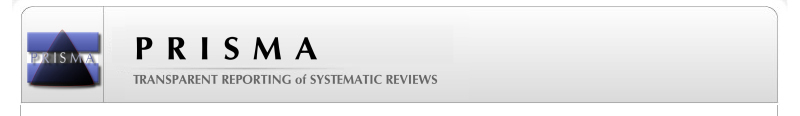
**Flowchart for how studies were identified in the rapid review methodology overview**

Studies included in qualitative synthesis
(n = 6)

Records identified through database searching
(n = 1358)

Full-text articles excluded

(n = 58)

Full-text articles assessed for eligibility
(n = 64)

Records excluded
(n = 1166)

Records screened
(n = 1230)

Records after duplicates removed
(n = 1230)

Additional records identified through other sources
(n = 4)

## Identification

## Eligibility

## Included

## Screening

c. Detailed results of the rapid review methodology overview

Six reviews were included in our our overview of reviews of streamlining methods. Two SRs examined the empirical evidence of abbreviating review methods and summarised the techniques in use. (Watt 2008, Ganann 2010) The third SR examined the descriptive and methodological characteristics of current OR practices, but did not address the implications of streamlining the processes (Pieper 2012). The three remaining reviews described a typology of reviews, and the use of rapid reviews in HTAs and OR methods. 21 strategies were formulated based on the SRs.

d. Detailed methods and results of the rapid NMA

*Protocol and registration*

To enable the comparison with a systematic review and network meta-analysis conducted in parallel by a different research team, we aligned the inclusion and exclusion criteria in our protocol to those in the research protocol of the systematic review. The rapid network meta-analysis was finalised in August 2014, whereas the systematic review and network meta-analysis was published in September 2014. All results of our rapid approach were completed prior to the gold standard NMA and reviewers were therefore blinded to the results in the systematic review and network meta-analysis.

*Eligibility criteria*

We considered randomised controlled trials in systematic reviews and in the McMasterPLUS database on the relative efficacy of pharmacological interventions on relapse and recurrence rates in bipolar disorder. We only included reviews with an available abstract, published electronically, in English, from 2006 onwards. The cut-off date was based on discussions with an expert in the field. Only randomised controlled trials comparing interventions with placebo or active comparator, included in the reviews, were eligible for data extraction. Quasi-randomised trials and continuation studies were excluded. No restrictions on settings were imposed. Authors of the original articles were not contacted for further information.

*Outcomes*

The number of participants with manic/mixed or depressive relapse or recurrence at the longest available follow-up.

*Participants*

Age 18 or older with a diagnosis of bipolar disorder type I or II, diagnosed according to DSM-III, DSM-IV-TR or ICD-10. Studies including patients with bipolar II only were excluded.

*Information sources*

We restricted our search for systematic reviews to the following electronic databases:

- Cochrane
- Medline
- EMBASE
- PsycINFO

In addition, we searched for randomised, controlled studies in the McMasterPLUS database. All searches were performed non-iteratively between September 1st and September 17th 2013. We tailored the search terms and filters to different databases. No grey literature databases were searched.

*Study selection*

A librarian performed the searches and removed duplicates. Two reviewers independently screened the references for SRs and applied eligibility criteria, based on title/abstract, and on the full text when necessary. Disagreements were solved through discussion.

*Assessment of risk of bias*

Two reviewers independently assessed the quality of the eligible SRs using the AMSTAR instrument. Disagreements on whether to include the studies were discussed until consensus. In addition, RCTs pre-appraised in the McMaster Knowledge Refinery were included. Risk of bias in the single studes was assessed using Cochrane criteria.

*Data collection process and data items*

A cumulative list of primary studies was constructed and duplicate studies removed. Two reviewers individually extracted data according to a minimum data extraction sheet developed by an experienced statistician and piloted on three articles. Data were extracted directly from the individual, independent primary studies in the SRs.

*Synthesis of results*

We used the arm-based, Bayesian NMAs method based on Markov Chain Monte Carlo simulation as described by Salanti. All multiple treatments meta-analyses were performed using Winbugs version 1.4.3 (Imperial College and MRC, UK). The statistical analysis was based on binomial likelihoods, with vague priors for the trial baselines, basic parameters and the random effects standard deviation, uniformly distributed in the interval 0 to 2. The analysis took the correlation structure induced by multi-arm trials into account. We utilised a random effects model following the normal distribution with 0 as the mean and standard deviation 0.0001. We expressed the comparative efficacy of the treatments as the relative risk (RR) of an outcome, with 95% credibility intervals (CrIs), and calculated SUCRAs. The credibility interval was the Bayesian analogue to confidence intervals used in traditional frequentist statistical approaches. We considered a result "significant" if the CrI did not include RR = 1. Single-standing nodes were excluded. The limitation of studies contributing to each network analysis was not quantified.

*Subgroups and sensitivity analysis*

To explore the effect of including both direct and indirect evidence in the analysis for the relative treatment effects, we performed sensitivity analyses, excluding indirect evidence for each pairwise comparison. These sensitivity analyses were performed in RevMan utilising random effects models. We planned to perform subgroup analyses for different disease types.

*The rapid network meta-analysis*

The combined search for SRs and McMasterPLUS primary studies identified 355 papers. 48 citations were retrieved in full text. We used the AMSTAR instrument to assess the quality of relevant SRs. Inconsistencies were solved by discussion with a third reviewer. All primary studies in the SRs could be extracted from two of the five reviews. After exclusion of primary studies in the SRs that did not meet the eligibility criteria, and inclusion of three pre-appraised studies in the McMasterPLUS database, 28 primary studies remained. Four studies were excluded because they were not a part of a network. Thus, 24 trials published between 1973 and 2012 were included in the NMA.

The included studies (Appendix 7) varied in size from 12 (reg Solomon) to 431 (ref Tohen 2005), and study duration ranged from six months to three years. The median rate of manic and depressive relapse was 29,2 (range 0 to 41 %) and 34,7 (range 14 to 100 %) in the ten available placebo controlled studies. The median rate of withdrawals from placebo groups due to reported side effects was 1.2 % (range 0 to 10 %) .

A total of 24 studies randomizing 3632 patients were available for the network meta-analysis. The studies covered 14 different treatment strategies. For all episodes, 9 of the 14 drugs were found to be statistically better than placebo, whereas for six options the difference was not significant. Only lithium was better than placebo in the prevention of both manic and depressive episodes.

e. Search strategy rapid NMA

*MeSH terms:*

(exp Bipolar disorder OR exp Depressive Disorder (Major depression, Recurrent depression used in PsychInfo)) AND (Drug Therapy OR Psychopharmacology) (+ Antidepressant Drugs used in PsychInfo)

*Filter strategies for identifying only network analysiss, meta-analyses and systematic reviews (1,2):*

We combined the strategies below with the MeSH terms to search the databases.

* = Truncation

[.tw= textword]

*Cochrane*

The databases ‘Cochrane Reviews’ and ‘Other Reviews’ were included as they only list meta-analyses and systematic reviews.

*Medline*

1. (systematic adj (review$1 or overview$1)).tw OR
2. meta analy$.tw OR
3. metaanaly$.tw OR
4. exp Meta-Analysis/ OR
5. (network adj1 (meta adj analy$) or metaanalys$).tw OR
6. (Multiple treatment comparison$).tw

*Embase:*

1. exp ‘SR’ OR
2. (meta adj analy$) or metaanalys$).tw OR
3. (network adj1 (meta adj analy$) or metaanalys$).tw OR
4. (systematic adj (review$1 or overview$1)).tw OR
5. (Multiple treatment comparison$).tw

*PsychINFO:*

1. exp Meta analysis/ OR
2. (network adj1 (meta adj analy$) or metaanalys$).tw OR
3. (systematic adj (review$1 or overview$1)).tw OR
4. (Multiple treatment comparison$).tw

*Primary studies in the McMaster PLUS database.*

Generic search strategy for searching the McMaster PLUS pre-appraised single studies:

(Bipolar disorder OR Depressive Disorder) AND

(Drug Therapy OR Psychopharmacology)

f. Flowchart for how studies were identified in the rapid NMA


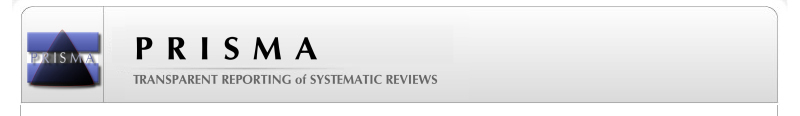
**Flowchart for how studies were identified in the rapid NMA**

Studies included in quantitative synthesis (network meta-analysis)
(n = 24)

Search for systematic reviews
(n = 353)

Full-text articles excluded, with reasons
(n = 24)

Full-text articles assessed for eligibility
(n = 48)

Records excluded
(n = 307)

Records screened
(n = 355)

Records after duplicates removed
(n = 355)

Records identified through McMasterPlus
(n = 2)

## Identification

## Eligibility

## Included

## Screening

g. AMSTAR evaluation of systematic reviews after assessment of relevance

|  | Soarez-W 2007 | Gallaegher 2008 | Ghaemi 2008 | Vasudev 2008 | Cipriani 2009 | Vieta 2011 |
| --- | --- | --- | --- | --- | --- | --- |
| **1. Was an 'a priori' design provided?** | Yes | Yes | Yes | Yes | Yes | Yes |
| **2. Was there duplicate study selection and data extraction?** | Yes | Yes | Yes | Yes | Yes | Two reviewers for data selection, no description of number involved in extraction |
| **3. Was a comprehensive literature search performed?** | Yes with some unclarity regarding consulting current contents. | Yes | Yes, supplemented by hand searches, reviews and contact with experts. Only keywords provided. | Yes | Yes | Only three databases searched. Reference lists searched. Rudimentary search strategy description. |
| **4. Was the status of publication (i.e. grey literature) used as an inclusion criterion?** | Grey literature searched and no restriction on language. No statement of publication status. | No restrictions. | No language restriction. Publication status not reason for exclusion. | No language restrictions. Reasons for exclusion of studies given. | No restrictions | Language restricted to English. No information on grey literature. |
| **5. Was a list of studies (included and excluded) provided?** | Yes | Yes | Yes | Yes | Yes | Yes |
| **6. Were the characteristics of the included studies provided?** | Most data provided in table. | Partly | Yes | Partly | Yes | Yes |
| **7. Was the scientific quality of the included studies assessed and documented?** | Yes | Yes | Yes | Yes | Yes | Not documented |
| **8. Was the scientific quality of the included studies used appropriately in formulating conclusions?** | N/A | N/A | N/A | N/A | N/A | N/A |
| **9. Were the methods used to combine the findings of studies appropriate?** | N/A | N/A | N/A | N/A | N/A | N/A |
| **10. Was the likelihood of publication bias assessed?** | N/A | N/A | N/A | N/A | N/A | N/A |
| **11. Was the conflict of interest stated?** | In the  systematic review | In the  systematic review | In the systematic  review | In the systematic review | In the  systematic review | In the systematic review |
| **Included in NMA?** | Yes | Yes | Yes | Yes | Yes | No |

Soares-Weiser K, Bravo Vergel Y, Beynon S, Dunn G, Barbieri M, Duffy S, et al. A systematic review and economic model of the clinical effectiveness and cost effectiveness of interventions for preventing relapse in people with bipolar disorder. Health Technol Assess. 2007;11(39):1-226.

Gallagher P, Malik N, Newham J, Young A, Ferrier IN, Mackin P. Antiglucocorticoid treatments for mood disorders, Cochrane Database Syst Rev 2008;CD005168.

Ghaemi SN, Wingo AP, Filkowski MA, Baldessarini RJ. Long-term antidepressant treatment in bipolar disorder: meta-analyses of benefits and risks. Acta Psychiatr Scand. 2008;118:347-356.

Vasudev A, Macritchie K, Watson S, Geddes JR, Young AH. Oxcarbazepine in the maintenance treatment of bipolar disorder. Cochrane Database Syst Rev 2008;CD005171.

Cipriani A, Rendell JM, Geddes J. Olanzapine in long-term treatment for bipolar disorder Cochrane Database Syst Rev 2009;CD004367.

Vieta E, Günther O, Locklear J, Ekman M, Miltenburger C, Chatterton ML et al. Effectiveness of psychotropic medications in the maintenance phase of bipolar disorder: a meta-analysis of randomized controlled trials. Int J Neuropsychopharmacol 2011;14:1029–49.

h. Minimum data extraction template

*A. Information regarding context of the study and intervention/control*

- What were the inclusion and exclusion criteria for participating in the study?
- In what context was the intervention and control implemented (primary/secondary/tertiary care; inpatient or outpatient; etc.)?
- Describe, as precisely as possible, the intervention
- Describe, as precisely as possible, the treatment given to control group participants
- For how long was the treatment given (calculated from baseline/time of randomisation)
- How long was the follow-up (calculated from baseline/time of randomisation)
- Were the groups similar at baseline (demographics, illness characteristics and prognostic factors)? YES/NO/UNCLEAR
- Which variables were tested for baseline similarity?

*B. Outcomes, analysis and results*

All available information is to be filled in (not all information will be available for all outcomes). If the absolute or relative effectiveness is based on re-analysis during the data extraction, then please describe the method used for re-analysis under ‘Description of the analysis performed’.

| **Outcome analysis and results** | **Article 1** | **Article 2** |
| --- | --- | --- |
| Outcome |  |  |
| Time (both relative to baseline and to end of treatment) |  |  |
| N intervention |  |  |
| Result Intervention (mean and std, or number of events) |  |  |
| N control |  |  |
| Result Control (mean and std, or number of events) |  |  |
| Absolute effect (intervention – control) including measure of uncertainty  (p-value, CI, SE of the mean) |  |  |
| Relative effect (intervention/control; RR, OR, HR) incl. measure of uncertainty  (p-value, CI, SE of the mean) |  |  |
| Description of the analysis performed |  |  |
| Adjusted for confounders? If yes, list the variables adjusted for in analysis |  |  |
| For continuous outcomes: Effect measured on the original scale  or as standardized effect (e.g.. Cohen’s d)? |  |  |

*C. Subgroup analyses*

Subgroups based on study/trial characteristics (results are the same as above):

| Characteristic used for subgroup analysis | The value for this study |
| --- | --- |
| Eg. setting | Tertiary care |
|  |  |

Subgroups based on participant characteristics (such as male/female, age, illness severity etc.):

For each sub-group the following table must be filled out:

SUBGROUP: ___________________________________________ (description)

| **Outcome analysis and results** | **Subgroup 1** | **Subgroup 2** |
| --- | --- | --- |
| Outcome |  |  |
| Time (both relative to baseline and to end of treatment) |  |  |
| N intervention |  |  |
| Result Intervention (mean and std, or number of events) |  |  |
| N control |  |  |
| Result Control (mean and std, or number of events) |  |  |
| Absolute effect (intervention – control) including measure of uncertainty  (p-value, CI, SE of the mean) |  |  |
| Relative effect (intervention/control; RR, OR, HR) including measure of uncertainty (p-value, CI, SE of the mean) |  |  |
| Was a separate analysis performed for the subgroup or was the subgroup results based on interactions from the main analysis? |  |  |

**Appendix 2: Relative risks of relapses**

| **ALL RELAPSES** | **RR ( RR 95% credible interval)   relative to placebo** | **SUCRA** |
| --- | --- | --- |
| Lithium+ divalproex | 0.03 (0.00 to 0.38) | 0,99 |
| Fluoxetine | 0.37 (0.09 to 0.88) | 0,84 |
| Olanzepine | 0.54 (0.43 to 0.68) | 0,75 |
| Lithium+ valproate | 0.54 (0.34 to 0.79) | 0,74 |
| Divalproex | 0.57 (0.39 to 0.79) | 0,70 |
| Aripipazole | 0.62 (0.37 to 0.99) | 0,60 |
| Lithium | 0.64 (0.53 to 0.75) | 0,58 |
| Valproate | 0.66 (0.42 to 0.92) | 0,55 |
| Lithium+ imipramine | 0.74 (0.49 to 1.11) | 0,43 |
| Risperidone LAI | 0.74 (0.51to 1.10) | 0,42 |
| Lithium+ amitriptyline | 0.83 (0.22 to 3.21) | 0,39 |
| Lamitrogine | 0.78 (0.61to 0.97) | 0,36 |
| Carbamazepine | 0.88 (0.61to 1.29) | 0,25 |
| Imipramine | 0.94 (0.68 to 1.29) | 0,20 |
| Placebo |  | 0,13 |
| Lithium, valproate, carbamazepine and perphenazine | 2.02 (0.50 to 10.42) | 0,08 |

SUCRA = surface under the cumulative ranking curve

| **MANIC/MIXED RELAPSES** | **RR (95% credible interval) relative to placebo** | **SUCRA** |
| --- | --- | --- |
| Olanzepine | 0.34 (0.22 to 0.51) | 0,86 |
| Risperidone LAI | 0.40 (0.19 to 0.77) | 0,77 |
| Lithium + valproate | 0.42 (0.20 to 0.82) | 0,75 |
| Aripipazole | 0.41 (0.16 to 0.92) | 0,74 |
| Lithium, valproate, carbamazepine and perphenazine | 0.27 (0.01to 2.67) | 0,73 |
| Lithium | 0.57 (0.39 to 0.79) | 0,58 |
| Divalproex | 0.64 (0.35 to 1.08) | 0,49 |
| Valproate | 0.68 (0.34 to 1.27) | 0,44 |
| Lamitrogine | 0.71 (0.44 to 1.11) | 0,42 |
| Lithium + amitriptyline | 0.76 (0.19 to 3.39) | 0,41 |
| Lithium + imipramine | 0.87 (0.41to 1.79) | 0,31 |
| Carbamazepine | 1.36 (0.26 to 12.95) | 0,21 |
| Placebo |  | 0,21 |
| Imipramine | 1.67 (0.89 to 3.17) | 0,06 |

| **DEPRESSIVE RELAPSES** | **RR (95% credible interval) relative to placebo** | **SUCRA** |
| --- | --- | --- |
| Imipramine | 0.39 (0.13 to 0.91) | 0,82 |
| Lithium + imipramine | 0.39 (0.13 to 0.96) | 0,81 |
| Fluoxetine | 0.36 (0.08 to 1.15) | 0,81 |
| Divalproex | 0.42 (0.19 to 0.88) | 0,79 |
| Lithium | 0.61(0.38 to 0.90) | 0,59 |
| Lithium + valproate | 0.61(0.22 to 1.38) | 0,59 |
| Lamitrogine | 0.67 (0.38 to 1.03) | 0,53 |
| Valproate | 0.72 (0.27 to 1.44) | 0,48 |
| Olanzepine | 0.80 (0.47 to 1.33) | 0,40 |
| Carbamazepine | 0.83 (0.32 to 2.09) | 0,38 |
| Aripipazole | 0.87 (0.29 to 2.56) | 0,38 |
| Placebo |  | 0,23 |
| Risperidone LAI | 1.28 (0.54 to 3.02) | 0,17 |
| Lithium, valproate, carbamazepine and perphenazine | 4.46 (0.68 to 54.51) | 0,04 |

**Appendix 3: Treatment burden results**

| **Option** | **Mean (SD)** | **Median (IR)** | **Min-max** |
| --- | --- | --- | --- |
| Basic burden common to all options | 32.1 (18.6) | 20.0 (30) | 7-70 |
| Valproate | 42.7 (23.8) | 32.6 (21) | 12-100 |
| Lamotrigine | 45.9 (26.6) | 37.5 (35) | 12-100 |
| Lamotrigine and valproate | 50.1 (23.6) | 45.0 (40) | 17-100 |
| Lithium | 53.1 (22.9) | 50.0 (31) | 11-100 |
| Imipramine | 54.0 (24.2) | 45.0 (38) | 12-100 |
| Carbamazepine | 56.2 (22.0) | 60.0 (33) | 16-100 |
| Lithium and oxcarbazepine | 57.4 (22.5) | 60.0 (30) | 14-100 |
| Lithium and valproate | 58.0 (21.4) | 60.0 (31) | 14-100 |
| Queatiapine | 58.3 (24.7) | 55.0 (48) | 14-100 |
| Aripiprazole | 61.9 (25.4) | 60.0 (39) | 14-100 |
| Lithium and imipramine | 61.9 (22.4) | 65.0 (31) | 14-100 |
| Olanzapine | 62.2 (26.0) | 60.0 (50) | 15-100 |
| Aripiprazole and valproate | 64.5 (24.8) | 65.0 (43) | 16-100 |
| Paliperidone | 66.4 (26.0) | 70.0 (46) | 16-100 |
| Aripiprazole and lamotrigine | 66.4 (25.2) | 65.0 (46) | 16-100 |
| Risperidone LAI | 70.7 (24.7) | 70.0 (41) | 17-100 |

“0” represents no treatment burden. IR=interquartile range. SD=standard deviation

**Appendix 4a: Questionnaire to physicians with experience in treating patients with bipolar disorder**

Provide an estimate: How many patients age 18 – 65 with bipolar disorder have you treated with each of the following treatment options during the last twenty years?

|  | 0-4 | 5-10 | 11-15 | 16-20 | 21-30 | 31-40 | 41-50 | 51-75 | 76-100 | > 100 |
| --- | --- | --- | --- | --- | --- | --- | --- | --- | --- | --- |
| Aripiprazole |  |  |  |  |  |  |  |  |  |  |
| Carbamazepine |  |  |  |  |  |  |  |  |  |  |
| Fluoxetine |  |  |  |  |  |  |  |  |  |  |
| Imipramine |  |  |  |  |  |  |  |  |  |  |
| Lithium |  |  |  |  |  |  |  |  |  |  |
| Lithium + Imipramine |  |  |  |  |  |  |  |  |  |  |
| Lithium + Oxcarbazepine |  |  |  |  |  |  |  |  |  |  |
| Lithium + Valproate |  |  |  |  |  |  |  |  |  |  |
| Lamotrigine |  |  |  |  |  |  |  |  |  |  |
| Aripirazole + Lamotrigine |  |  |  |  |  |  |  |  |  |  |
| Valproate + Lamotrigine |  |  |  |  |  |  |  |  |  |  |
| Olanzapine |  |  |  |  |  |  |  |  |  |  |
| Paliperidone |  |  |  |  |  |  |  |  |  |  |
| Quetiapine |  |  |  |  |  |  |  |  |  |  |
| Risperidone LAI |  |  |  |  |  |  |  |  |  |  |
| Valproate |  |  |  |  |  |  |  |  |  |  |
| Valproate + Aripiprazole |  |  |  |  |  |  |  |  |  |  |
| Other |  |  |  |  |  |  |  |  |  |  |
| No medicines |  |  |  |  |  |  |  |  |  |  |

For all the medicines/combinations of medicines you have used to treat more than five patients: provide a percentage in each of the next questions (see your own list above).

**Percentage with ACUTE MANIC EPISODES**

Provide an estimate based on your experience, for each treatment option: How many of your patients with bipolar disorder (age 18 – 65) experience at least one, manic episode during a period of time of 2 years? For each treatment option, only provide a percentage if you have treated more than five patients.

|  | 10  % | 20  % | 30  % | 40  % | 50  % | 60  % | 70  % | 80  % | 90  % | 100  % |
| --- | --- | --- | --- | --- | --- | --- | --- | --- | --- | --- |
| Aripiprazole |  |  |  |  |  |  |  |  |  |  |
| Carbamazepine |  |  |  |  |  |  |  |  |  |  |
| Fluoxetine |  |  |  |  |  |  |  |  |  |  |
| Imipramine |  |  |  |  |  |  |  |  |  |  |
| Lithium |  |  |  |  |  |  |  |  |  |  |
| Lithium + Imipramine |  |  |  |  |  |  |  |  |  |  |
| Lithium + Oxcarbazepine |  |  |  |  |  |  |  |  |  |  |
| Lithium + Valproate |  |  |  |  |  |  |  |  |  |  |
| Lamotrigine |  |  |  |  |  |  |  |  |  |  |
| Aripirazole + Lamotrigine |  |  |  |  |  |  |  |  |  |  |
| Valproate + Lamotrigine |  |  |  |  |  |  |  |  |  |  |
| Olanzapine |  |  |  |  |  |  |  |  |  |  |
| Paliperidone |  |  |  |  |  |  |  |  |  |  |
| Quetiapine |  |  |  |  |  |  |  |  |  |  |
| Risperidone LAI |  |  |  |  |  |  |  |  |  |  |
| Valproate |  |  |  |  |  |  |  |  |  |  |
| Valproate + Aripiprazole |  |  |  |  |  |  |  |  |  |  |
| Other |  |  |  |  |  |  |  |  |  |  |
| No medicines |  |  |  |  |  |  |  |  |  |  |

**Percentage with acute depressive episodes**

Make an estimate based on your experience, for each treatment option: How many of your patients with bipolar disorder (age 18 – 65) experience at least one, depressive episode, during a period of time of 2 years? For each treatment option, only provide a percentage if you have treated more than five patients.

|  | 10  % | 20  % | 30  % | 40  % | 50  % | 60  % | 70  % | 80  % | 90  % | 100  % |
| --- | --- | --- | --- | --- | --- | --- | --- | --- | --- | --- |
| Aripiprazole |  |  |  |  |  |  |  |  |  |  |
| Carbamazepine |  |  |  |  |  |  |  |  |  |  |
| Fluoxetine |  |  |  |  |  |  |  |  |  |  |
| Imipramine |  |  |  |  |  |  |  |  |  |  |
| Lithium |  |  |  |  |  |  |  |  |  |  |
| Lithium + Imipramine |  |  |  |  |  |  |  |  |  |  |
| Lithium + Oxcarbazepine |  |  |  |  |  |  |  |  |  |  |
| Lithium + Valproate |  |  |  |  |  |  |  |  |  |  |
| Lamotrigine |  |  |  |  |  |  |  |  |  |  |
| Aripirazole + Lamotrigine |  |  |  |  |  |  |  |  |  |  |
| Valproate + Lamotrigine |  |  |  |  |  |  |  |  |  |  |
| Olanzapine |  |  |  |  |  |  |  |  |  |  |
| Paliperidone |  |  |  |  |  |  |  |  |  |  |
| Quetiapine |  |  |  |  |  |  |  |  |  |  |
| Risperidone LAI |  |  |  |  |  |  |  |  |  |  |
| Valproate |  |  |  |  |  |  |  |  |  |  |
| Valproate + Aripiprazole |  |  |  |  |  |  |  |  |  |  |
| Other |  |  |  |  |  |  |  |  |  |  |
| No medicines |  |  |  |  |  |  |  |  |  |  |

**Percentage with MANIC SYMPTOMS between episodes**

Provide an estimate based on your experience: How many of your patients (age 18 – 65) with bipolar disorder are mildly or moderately manic at least half of all days between acute manic or depressive episodes during a 2-years period of time? For each treatment option, only provide an estimate if you have treated at least five patients with the treatment option.

|  | 10  % | 20  % | 30  % | 40  % | 50  % | 60  % | 70  % | 80  % | 90  % | 100  % |
| --- | --- | --- | --- | --- | --- | --- | --- | --- | --- | --- |
| Aripiprazole |  |  |  |  |  |  |  |  |  |  |
| Carbamazepine |  |  |  |  |  |  |  |  |  |  |
| Fluoxetine |  |  |  |  |  |  |  |  |  |  |
| Imipramine |  |  |  |  |  |  |  |  |  |  |
| Lithium |  |  |  |  |  |  |  |  |  |  |
| Lithium + Imipramine |  |  |  |  |  |  |  |  |  |  |
| Lithium + Oxcarbazepine |  |  |  |  |  |  |  |  |  |  |
| Lithium + Valproate |  |  |  |  |  |  |  |  |  |  |
| Lamotrigine |  |  |  |  |  |  |  |  |  |  |
| Aripirazole + Lamotrigine |  |  |  |  |  |  |  |  |  |  |
| Valproate + Lamotrigine |  |  |  |  |  |  |  |  |  |  |
| Olanzapine |  |  |  |  |  |  |  |  |  |  |
| Paliperidone |  |  |  |  |  |  |  |  |  |  |
| Quetiapine |  |  |  |  |  |  |  |  |  |  |
| Risperidone LAI |  |  |  |  |  |  |  |  |  |  |
| Valproate |  |  |  |  |  |  |  |  |  |  |
| Valproate + Aripiprazole |  |  |  |  |  |  |  |  |  |  |
| Other |  |  |  |  |  |  |  |  |  |  |
| No medicines |  |  |  |  |  |  |  |  |  |  |

**Percentage with DEPRESSIVE SYMPTOMS between episodes**

Provide an estimate based on your experience: How many of your patients (age 18 – 65) with bipolar disorder are mildly or moderately depressive at least half of all days between acute manic or depressive episodes during a 2-years period of time? For each treatment option, only provide an estimate if you have treated at least five patients with the treatment option.

|  | 10  % | 20  % | 30  % | 40  % | 50  % | 60  % | 70  % | 80  % | 90  % | 100  % |
| --- | --- | --- | --- | --- | --- | --- | --- | --- | --- | --- |
| Aripiprazole |  |  |  |  |  |  |  |  |  |  |
| Carbamazepine |  |  |  |  |  |  |  |  |  |  |
| Fluoxetine |  |  |  |  |  |  |  |  |  |  |
| Imipramine |  |  |  |  |  |  |  |  |  |  |
| Lithium |  |  |  |  |  |  |  |  |  |  |
| Lithium + Imipramine |  |  |  |  |  |  |  |  |  |  |
| Lithium + Oxcarbazepine |  |  |  |  |  |  |  |  |  |  |
| Lithium + Valproate |  |  |  |  |  |  |  |  |  |  |
| Lamotrigine |  |  |  |  |  |  |  |  |  |  |
| Aripirazole + Lamotrigine |  |  |  |  |  |  |  |  |  |  |
| Valproate + Lamotrigine |  |  |  |  |  |  |  |  |  |  |
| Olanzapine |  |  |  |  |  |  |  |  |  |  |
| Paliperidone |  |  |  |  |  |  |  |  |  |  |
| Quetiapine |  |  |  |  |  |  |  |  |  |  |
| Risperidone LAI |  |  |  |  |  |  |  |  |  |  |
| Valproate |  |  |  |  |  |  |  |  |  |  |
| Valproate + Aripiprazole |  |  |  |  |  |  |  |  |  |  |
| Other |  |  |  |  |  |  |  |  |  |  |
| No medicines |  |  |  |  |  |  |  |  |  |  |

**Appendix 4b: Experts´ ranking and effect estimates for maintenance treatments, compared to results from the systematic review: depressive episodes.**

| **Experts (n=10) mean ranking of treatment options for the outcome ‘avoid acute manic episodes’**  **(mean percentage avoiding acute manic episodes, SUCRA value)** |  | **SR (n=33) mean ranking of treatment options for the outcome ‘avoid acute manic episodes’**  **(mean percentage avoiding acute manic episodes, SUCRA value)** |
| --- | --- | --- |
| Paliperidone (86), 0.85 |  | Olanzapine (91) |
| Lithium + Valproate (80), 0.79 |  | Litihum + Oxcarbazepine (90) |
| Valproate + Aripiprazole (78), 0.66 |  | Litihum + Valproate (88) |
| Imipramine (78), 0.64 |  | Risperidone (88) |
| Risperidone (76), 0.58 |  | Aripiprazole + Lamotrigine (86) |
| Litihum + Oxcarbazepine (76), 0.55 |  | Aripiprazole (86) |
| Carbamazepine (76), 0.58 |  | Valproate + Aripiprazole (85) |
| Valproate + Lamotrigine (75), 0.54 |  | Litihum (84) |
| Lithium + Imipramine (75), 0.51 |  | Paliperidone (83) |
| Valproate (74), 0.52 |  | Quetiapine (83) |
| Aripirazole + Lamotrigine (74), 0.51 |  | Valproate (82) |
| Olanzapine (73), 0.44 |  | Lithium + Imipramine (78) |
| Aripiprazole (72), 0.39 |  | Lamotrigine (75) |
| Lithium (72), 0.38 |  | **No medication (72)** |
| Quetiapine (71), 0.35 |  | Imipramine (64) |
| No medication (70), 0.34 |  | Carbamazepine (54) |
| Lamotrigine (67), 0.16 |  | Valproate + Lamotrigine (52) |

*Kendall´s tau b rank correlation: 0.118 (p=0.51)*

**Appendix 4c: Experts´ ranking and effect estimates for maintenance treatments, compared to results from the systematic review: manic episodes.**

| **Experts (n=10) mean ranking of treatment options for the outcome ‘avoid acute manic episodes’**  **(mean percentage avoiding acute manic episodes, SUCRA value)** |  | **SR (n=33) mean ranking of treatment options for the outcome ‘avoid acute manic episodes’**  **(mean percentage avoiding acute manic episodes, SUCRA value)** |
| --- | --- | --- |
| Paliperidone (86), 0.85 |  | Olanzapine (91) |
| Lithium + Valproate (80), 0.79 |  | Litihum + Oxcarbazepine (90) |
| Valproate + Aripiprazole (78), 0.66 |  | Litihum + Valproate (88) |
| Imipramine (78), 0.64 |  | Risperidone (88) |
| Risperidone (76), 0.58 |  | Aripiprazole + Lamotrigine (86) |
| Litihum + Oxcarbazepine (76), 0.55 |  | Aripiprazole (86) |
| Carbamazepine (76), 0.58 |  | Valproate + Aripiprazole (85) |
| Valproate + Lamotrigine (75), 0.54 |  | Litihum (84) |
| Lithium + Imipramine (75), 0.51 |  | Paliperidone (83) |
| Valproate (74), 0.52 |  | Quetiapine (83) |
| Aripirazole + Lamotrigine (74), 0.51 |  | Valproate (82) |
| Olanzapine (73), 0.44 |  | Lithium + Imipramine (78) |
| Aripiprazole (72), 0.39 |  | Lamotrigine (75) |
| Lithium (72), 0.38 |  | **No medication (72)** |
| Quetiapine (71), 0.35 |  | Imipramine (64) |
| No medication (70), 0.34 |  | Carbamazepine (54) |
| Lamotrigine (67), 0.16 |  | Valproate + Lamotrigine (52) |

*Kendall´s tau b rank correlation: 0.118 (p=0.51)*

**_______________________________________________________________________________________**

**Stage two: impact on expected values and rankings of treatments of rapid and gold standard methods______________________________________________**

**Appendix 5: Patients´ relative importance weights**

| **Outcome** | **Mean SEA scores:  Mean (SD), Median (IR), [min-max]** |
| --- | --- |
| Avoid severe depressive episodes | 78 (26), 83 (17), [13-100] |
| Avoid severe manic episodes | 66 (30), 67 (56), [7-100] |
| Avoid side effects | 40 (19), 40 (32), [13-83] |
| Avoid mild depression between acute episodes | 34 (17), 35 (20), [7-83] |
| Avoid mild mania between acute episodes | 21 (12), 20 (16), [3-53] |
| Avoid treatment burden | 15 (14), 8 (17), [3-40] |

SEA: self-explicated approach; SD: standard deviation; IR: interquartile range

**Appendix 6 : Descriptive comparison of the rapid review and systematic review**

*Outcomes*

Both the rapid and the systematic NMA included any mood episode relapse, manic/mixed episode relapse, and depressive episode relapse. The systematic review also included tolerability and acceptability in the network analysis.

*Included trials*

We identified and included 24 randomized controlled trials in the rapid NMA, in contrast to the 33 in the gold standard NMA. 19 trials were included in both reviews. The gold standard NMA included 14 trials that had not been identified in our rapid process. 5 papers in the rapid NMA were not included in the systematic NMA.

*Included options*

Treatment options included in both the rapid and the gold standard NMA included aripiprazole, carbamazepine, fluoxetine, imipramine, lamotrigine, lithium, lithium + imipramine, lithium + valproate, olanzapine, placebo, risperidone LAI, and valproate. The gold standard NMA additionally included paliperidone (the primary active metabolite of risperidone), quetiapine, aripiprazole+valproate, lithium + oxcarbazepine, and valproate + lamotrigine, neither of which were included in the rapid NMA. The rapid NMA included two medications not included in the gold standard NMA, i.e. pherpenazine and amitriptyline.

*Efficacy of the options*

The rapid NMA included 8 of the 9 treatments and treatment combinations included in the closed loop network presented in the gold standard NMA, quetiapine being the exception. For the 8 overlapping treatments, the conclusion on whether the treatment significantly reduced the risk of all, manic/mixed and depressive episodes was similar for 6, 8 and 5 treatments, respectively. The rapid NMA included 5 treatments in the closed loop network which were not in the closed loop network of the gold standard NMA.

*Assessment of risk of bias*

In the gold standard NMA, the risk of bias for each included trial was assessed. In the rapid NMA we avoided this step by summarising the assessments of risk of bias available in the systematic reviews into one overall assessment. Below are the risks of bias stated in the gold standard NMA and the overall assessments of risks of bias in the rapid NMA. With the possible exeption of Coxhead 1992, the assessments in the rapid and gold standard NMA are comparable.


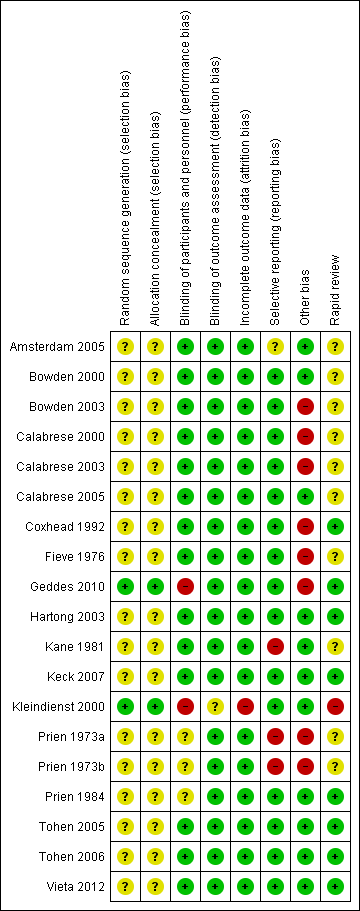


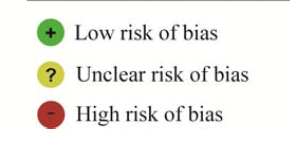


The figure shows the distribution of risks of bias for the studies in the systematic review and the overall assessment in the rapid review. The rightmost column presents the assessments in the rapid review; all other assessments were presented in the systematic review.

*Subgroup analysis*

Neither the rapid review nor the systematic review performed a separate analysis for bipolar disorder type II, due to lack of evidence. The systematic review performed a separate analysis for bipolar type I.

*Reasons for discrepancy of results*

Five principal reasons for the discrepancy of results were identified. First, our search strategy failed to identify studies in the systematic review that had been published after the search date of the most recent systematic review, published in 2010. The two included studies had been identified by the McMaster PLUS service. Second, three of the studies included in the systematic review were excluded in the rapid review because of differing inclusion criteria. Third, three studies included in the five systematic reviews were excluded by the rapid review reviewers due to risk of bias. A forth reason for discrepancy between the rapid review and the systematic review was the systematic review defining the two options divalproex and valproate as identical treatments, whereas in the rapid review these two medications were treated as separate modalities. Fifth, discarding all studies in a systematic review based on low AMSTAR scores of the systematic review in which it was included, resulted in the non-inclusion of two studies in the rapid review, that were included in the systematic review.

**Appendix 7: PRISMA 2009 Checklist**

| **Section/topic** | **#** | **Checklist item** | **Reported on page #** |
| --- | --- | --- | --- |
| **TITLE** | | |  |
| Title | 1 | Identify the report as a systematic review, meta-analysis, or both. | p. 1 |
| **ABSTRACT** | | |  |
| Structured summary | 2 | Provide a structured summary including, as applicable: background; objectives; data sources; study eligibility criteria, participants, and interventions; study appraisal and synthesis methods; results; limitations; conclusions and implications of key findings; systematic review registration number. | pp. 3, 4, appendix 1d pp. 4 - 5 |
| **INTRODUCTION** | | |  |
| Rationale | 3 | Describe the rationale for the review in the context of what is already known. | pp. 3 - 5 |
| Objectives | 4 | Provide an explicit statement of questions being addressed with reference to participants, interventions, comparisons, outcomes, and study design (PICOS). | pp. 8 – 10, appendix 1d pp. 4 - 5 |
| **METHODS** | | |  |
| Protocol and registration | 5 | Indicate if a review protocol exists, if and where it can be accessed (e.g., Web address), and, if available, provide registration information including registration number. | Protocol exists but not published |
| Eligibility criteria | 6 | Specify study characteristics (e.g., PICOS, length of follow-up) and report characteristics (e.g., years considered, language, publication status) used as criteria for eligibility, giving rationale. | Table 6, 34 – 35. appendix 1d pp. 4 - 5 |
| Information sources | 7 | Describe all information sources (e.g., databases with dates of coverage, contact with study authors to identify additional studies) in the search and date last searched. | Table 6, 34 – 35. appendix 1d pp. 4 - 5 |
| Search | 8 | Present full electronic search strategy for at least one database, including any limits used, such that it could be repeated. | Appendix 1e, p. 6 |
| Study selection | 9 | State the process for selecting studies (i.e., screening, eligibility, included in systematic review, and, if applicable, included in the meta-analysis). | pp. 8 – 9, appendix 1d pp. 4 - 5 |
| Data collection process | 10 | Describe method of data extraction from reports (e.g., piloted forms, independently, in duplicate) and any processes for obtaining and confirming data from investigators. | Appendix 1d pp. 4 - 5, appendix 1h, pp. 10 - 11 |
| Data items | 11 | List and define all variables for which data were sought (e.g., PICOS, funding sources) and any assumptions and simplifications made. | Appendix 1d pp. 4 – 5, appendix 1h, pp. 10 - 11 |
| Risk of bias in individual studies | 12 | Describe methods used for assessing risk of bias of individual studies (including specification of whether this was done at the study or outcome level), and how this information is to be used in any data synthesis. | Appendix 1d, p. 5 |
| Summary measures | 13 | State the principal summary measures (e.g., risk ratio, difference in means). | pp. 8 – 9, appendix 1h pp. 10 - 11 |
| Synthesis of results | 14 | Describe the methods of handling data and combining results of studies, if done, including measures of consistency (e.g., I^2^) for each meta-analysis. | Appendix 1d, p. 5 |

Page 1 of 2

| **Section/topic** | **#** | **Checklist item** | **Reported on page #** |
| --- | --- | --- | --- |
| Risk of bias across studies | 15 | Specify any assessment of risk of bias that may affect the cumulative evidence (e.g., publication bias, selective reporting within studies). | Robustness of results estimated in sensitivity analyses of MCDA pp. 19 – 22. |
| Additional analyses | 16 | Describe methods of additional analyses (e.g., sensitivity or subgroup analyses, meta-regression), if done, indicating which were pre-specified. | Appendix 1d, p. 5 |
| **RESULTS** | | |  |
| Study selection | 17 | Give numbers of studies screened, assessed for eligibility, and included in the review, with reasons for exclusions at each stage, ideally with a flow diagram. | Appendix 1f, p. 7 |
| Study characteristics | 18 | For each study, present characteristics for which data were extracted (e.g., study size, PICOS, follow-up period) and provide the citations. | Available on request |
| Risk of bias within studies | 19 | Present data on risk of bias of each study and, if available, any outcome level assessment (see item 12). | Appendix 6, pp. 21 - 22 |
| Results of individual studies | 20 | For all outcomes considered (benefits or harms), present, for each study: (a) simple summary data for each intervention group (b) effect estimates and confidence intervals, ideally with a forest plot. | Available on request |
| Synthesis of results | 21 | Present results of each meta-analysis done, including confidence intervals and measures of consistency. | Appendix 2, pp. 12 - 13 |
| Risk of bias across studies | 22 | Present results of any assessment of risk of bias across studies (see Item 15). | Robustness of results estimated in sensitivity analyses of MCDA pp. 19 – 22. |
| Additional analysis | 23 | Give results of additional analyses, if done (e.g., sensitivity or subgroup analyses, meta-regression [see Item 16]). | Sensitivity analyses pp. 19 – 20. |
| **DISCUSSION** | | |  |
| Summary of evidence | 24 | Summarize the main findings including the strength of evidence for each main outcome; consider their relevance to key groups (e.g., healthcare providers, users, and policy makers). | pp. 23 - 29 |
| Limitations | 25 | Discuss limitations at study and outcome level (e.g., risk of bias), and at review-level (e.g., incomplete retrieval of identified research, reporting bias). | pp. 26 – 27, appendix 6, pp. 18 ­– 19 |
| Conclusions | 26 | Provide a general interpretation of the results in the context of other evidence, and implications for future research. | pp. 23 - 29 |
| **FUNDING** | | |  |
| Funding | 27 | Describe sources of funding for the systematic review and other support (e.g., supply of data); role of funders for the systematic review. | p. 31 |

*From:*  Moher D, Liberati A, Tetzlaff J, Altman DG, The PRISMA Group (2009). Preferred Reporting Items for Systematic Reviews and Meta-Analyses: The PRISMA Statement. PLoS Med 6(6): e1000097. doi:10.1371/journal.pmed1000097

For more information, visit: **www.prisma-statement.org**.

Page 2 of 2

**Appendix 8: Comparison of methods based on PRISMA**

| **METHODS** | **Rapid review** | **Systematic review** | **Substantial difference** |
| --- | --- | --- | --- |
| Protocol and registration | Protocol developed but not published | Published in PROSPERO database. Corrections published. | Yes |
| **Adherence to PRISMA criteria** | Pragmatically | Fully | Yes |
| Eligibility criteria |  |  |  |
| **Study design** | RCTs and open RCTs from systematic reviews and from the McMaster database | RCTs and open RCTs. | No |
| **Publication date** | Systematic reviews published since 2006 and RCTs published after the search date of the most recent review | RCTs and open RCTs published since inception of databases | Yes |
| **Language** | English only | No language restrictions | Yes |
| **Publication status** | Easily retrievable abstract and full-text, electronic sources only | No restrictions | Yes |
| **Follow-up** | At least 3 months | At least 12 weeks | No |
| **Diagnosis** | Bipolar disorder type I and II | Bipolar disorder irrespective of specification of subtype | Yes |
| **Monotherapy or add-on treatment** | Both | Both | No |
| **Age** | 18 and older | 18 and older | No |
| **Blinding** | Any level of blinding included | Any level of blinding included | No |
| Information sources |  |  |  |
| **Databases** | *Systematic reviews:*  Cochrane reviews  Cochrane, other  Medline  EMBASE  PsycINFO  *RCTs:*  McMasterPLUS | *RCTs:*  Embase  Medline  PreMedline  PsycINFO  CENTRAL  ICTRP  FDA | Yes |
| **Contact with authors** | No | Yes | Yes |
| **Last date of search** | October 29th 2013 | July 4th 2013 | Yes |
| Search |  |  |  |
|  | Full search strategy submitted for publication | Full search strategy published | No |
| Study selection |  |  |  |
| **Reviewers** | Two independent reviewers | Two independent reviewers | No |
| **Exclusion** | Continuation studies | 1. Continuation studies.  2. Lithium or valproate as baseline treatment in combination/augmentation studies | Yes |
| Data collection process |  |  |  |
| **Two reviewers** | Two independent reviewers | Two independent reviewers | No |
| **Obtaining and confirming data from investigators** | No | When necessary | Yes |
| Data items |  |  |  |
| **Primary outcomes, treatment efficacy** | Number of patients in intervention and control group with any, manic or depressive episode. | Number of any mood episode (depressive, manic or mixed relapse) as defined by author at the longest available follow-up. | Not for treatment efficacy |
| **Risk of bias in individual studies** | AMSTAR criteria were applied to systematic reviews.  Expedite assessment of individual studies applying principles from the Cochrane collaboration. | Cochrane collaboration method and GRADE | Yes |
| **Summary measures** | Relative risk with 95 % credible intervals,  SUCRA | Risk ratio with 95 % credible intervals,  SUCRA | No |
| Methods for combining results |  |  |  |
| **Assessment of risk of bias that may affect cumulative evidence** | Not quantified | Limitiation of studies contributing to each network estimate quantified | Yes |
| **Blinding** | Blinded drugs, open-label drugs and blinded placebo allowed in same drug node in network meta-analysis | Blinded drugs, open-label drugs and blinded placebo allowed in same drug node in network meta-analysis | No |
| **Network meta-analysis** | Random effects network meta-analysis within a Bayesian framework using Markov chain Monte Carlo | Random effects network meta-analysis within a Bayesian framework using Markov chain Monte Carlo | No |
| **Analysis software** | Winbugs 1.4.3 | OpenBUGS 3.2.2 | No |
| **Sensitivity analysis** | Sensitivity analyses with respect to bipolar disorder subtype was planned but not effectuated due to insufficient data | Sensitivity analysis according to publication year, subtypes of bipolar disorder, rapid-cycling course of illness, enrichment design, sponsorship bias, duration of follow-up, and blinding of the treatment group. The analyses mainly focused on the treatment nodes constituting the closed-loop network. | Yes |

**____________________________________________________________________**

**Discussion**

**_______________________________________________________________________________________**

**Appendix 9: Importance weights and patient involvement in MCDAs**

| Study (n=10) | **Importance weights:** averages, individual or other? | **Source of outcome:**  Patients only, patients to some degree, not patients? |
| --- | --- | --- |
| Brockhuizen 2012* |  | Not patients |
| Airoldi 2011 | Likely average per person | Patients to some degree (n=3) |
| Hummel 2012 | Group average (mean of all pairwise comparisons) | Not patients |
| Dolan 2005 | Group mean/median values, but analysis of variance also performed | Not patients |
| Hummel 2005 | Group average | Patients to some degree (n=34) |
| Goetgehebeur 2010 | Group average | Not patients |
| Youngkong 2012 | Group average | Patients to some degree (n=6) |
| Sussex 2013 | Group average | Patients to some degree (several workshops with 6-11 patients in each) |
| Dolan 2013 | Group average | Not patients |
| Hummel 2013 | Group average | Not patients |

*) Original article not found.
